# *In situ* mass spectrometry analysis of intact proteins and protein complexes from biological substrates

**DOI:** 10.1042/BST20190793

**Published:** 2020-02-03

**Authors:** Oliver J. Hale, Helen J. Cooper

**Affiliations:** School of Biosciences, University of Birmingham, Edgbaston B15 2TT, U.K.

**Keywords:** ambient, *in situ*, mass spectrometry, native, protein

## Abstract

Advances in sample preparation, ion sources and mass spectrometer technology have enabled the detection and characterisation of intact proteins. The challenges associated include an appropriately soft ionisation event, efficient transmission and detection of the often delicate macromolecules. Ambient ion sources, in particular, offer a wealth of strategies for analysis of proteins from solution environments, and directly from biological substrates. The last two decades have seen rapid development in this area. Innovations include liquid extraction surface analysis, desorption electrospray ionisation and nanospray desorption electrospray ionisation. Similarly, developments in native mass spectrometry allow protein–protein and protein–ligand complexes to be ionised and analysed. Identification and characterisation of these large ions involves a suite of hyphenated mass spectrometry techniques, often including the coupling of ion mobility spectrometry and fragmentation techniques. The latter include collision, electron and photon-induced methods, each with their own characteristics and benefits for intact protein identification. In this review, recent developments for *in situ* protein analysis are explored, with a focus on ion sources and tandem mass spectrometry techniques used for identification.

## Introduction

Analysis of proteins *in situ* has become a focus in the development of many mass spectrometer technologies, especially ion sources. *In situ* analysis is attractive as information may be obtained rapidly, with little to no sample preparation. Consequently, many developments have been made with robotic systems. A further benefit is the opportunity to analyse proteins directly from their physiological environment (body fluids, tissue, etc.), which allows spatial information about the protein distribution to be recorded, so-called mass spectrometry imaging (MSI). MSI has typically been the realm of matrix-assisted laser desorption/ionisation (MALDI) MS [1], an ionisation technique that is usually performed under vacuum conditions; however, MALDI MSI generally requires complementary methods to identify the proteins detected since MALDI-generated protein ions are predominantly in low charge states and are thus not amenable to efficient fragmentation for top-down identification [2]. The high *m*/*z* of the MALDI-generated protein ions also necessitates the use of specific, high *m*/*z* capable mass spectrometers, which are not necessary for ionisation techniques that produce highly charged ions. As such, developments in soft and ambient ionisation techniques that do produce highly charged ions are now showing their value for intact protein analysis, and without the time-consuming sample preparation required for MALDI.

Identification of proteins may be performed in a ‘top-down' methodology, whereby the intact proteins are ionised and then fragmented within the mass spectrometer to provide sequence information. The fragments generated depend on the fragmentation technique used. Protein identification is achieved by automated searching of the fragment *m*/*z* values against protein databases. Alternatively, digestion by specific enzymes, such as trypsin, enable ‘bottom-up’ proteomics; the peptides produced by protein digestion are analysed and identified, then the source protein is determined by matching to sequences in a database. On-tissue digestion followed by MALDI MS allows for bottom-up protein identification from peptides but generally results in some loss of integrity of protein spatial distribution. Bottom-up proteomics may also result in missing information such as the connectivity of labile post-translational modifications (PTMs), presence of single-nucleotide polymorphisms and ambiguity as to the source of redundant peptides [3]. Multiple alternatives to MALDI for *in situ* protein analysis now exist, many taking advantage of the highly charged ions produced by electrospray ionisation (ESI)-like processes. This review covers these alternative techniques and related technologies that allow for *in situ* protein detection and identification.

## Ambient ionisation

*In situ* mass spectrometry analysis has benefitted from the many ambient ionisation techniques that have been developed in the last two decades. The analysis is performed at atmospheric pressure and usually without the constraints on sample size and sample state imposed by *in vacuo* ion sources. Here, we focus on those techniques that have been applied to the analysis of intact proteins.

## Liquid junction surface sampling

Many liquid extraction-based ion sources for mass spectrometry are described in the literature, many leveraging the flexibility and sensitivity of solvent-based extraction with subsequent ESI. ESI has a 30-year heritage of protein analysis [5,6] and resulted in one-quarter of the Nobel Prize in Chemistry in 2002. It was later developed into nanoESI, dramatically reducing droplet size, improving sensitivity and salt tolerance at a much-reduced sample consumption rate [7]. NanoESI is the major form of the technique used for biomolecule analysis today. The various liquid extraction techniques include liquid extraction surface analysis (LESA) [8], nanospray desorption electrospray ionisation (nano-DESI) and devices such as the FlowProbe (Prosolia Inc. Indianapolis, IN) and the MasSpec Pen [9]. In the last decade, these techniques have become established for analysis of small molecules, metabolites and lipids directly from biological samples (LESA [10–12], nano-DESI [13–15], FlowProbe [16], MasSpec Pen [17]), but *in situ* protein analysis has proven a greater challenge.

## Liquid extraction surface analysis (LESA)

**Figure 1.**
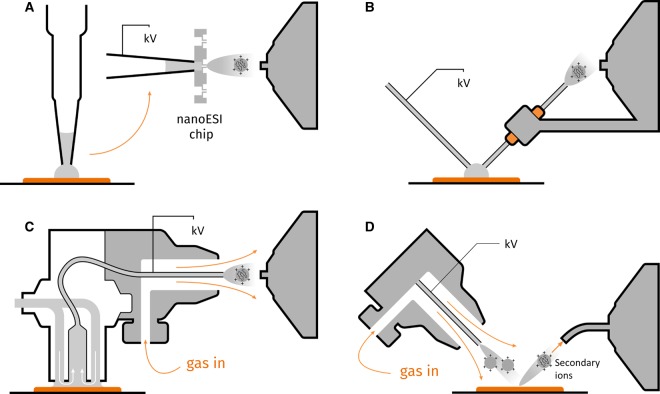
Schematics of ambient ion sources. (**A**) LESA, (**B**) nano-DESI, (**C**) FlowProbe and (**D**) DESI. Adapted from reference [4] (DOI: 10.1002/jms.4087) under the Creative Commons Attribution Licence (CC-BY) (http://creativecommons.org/licenses/by/4.0/).

LESA (Figure 1A) is perhaps the most established liquid extraction method for *in situ* protein analysis, and was successfully commercialised in a robotic sampling system by Advion (Ithaca, NY) building on work by Kertesz et al. [8] on liquid microjunction surface sampling probes (LMJ-SSP). Other LESA robotic systems have been developed, enabling online chromatographic separation, laser-guided sampling probe height and more rapid sampling [18,19]. Since LESA is a commercially available, automatable technique based on the robustness of ESI, and requires little to no sample preparation, it presents the most accessible *in situ* analysis technique considered here.

Typically, LESA is performed by dispensing solvent from a pipette tip to form a liquid junction between the tip and the sample surface, allowing analytes to dissolve. A variant of the technique is contact LESA [20], in which the pipette tip maintains contact with the sample surface during extraction, confining the extraction solvent and sampling a smaller area. Contact LESA enables improved spatially-specific sampling since an area ∼400 μm in diameter is sampled, compared with ∼1 mm for LESA. After a defined period (seconds), the solvent is aspirated back into the pipette tip, which is then moved to the mass spectrometer inlet for nanoESI. Direct coupling to high-performance liquid chromatography for analyte separation and concentration prior to mass analysis is also possible and can be performed with commercial systems [18,21]. Recent bottom-up and top-down proteomics studies have been performed in this way and provide complementary data to conventional MSI experiments, with higher spatial resolution (∼110 μm) recently achieved with the microLESA setup [22–24]. Since the spatial resolution is largely governed by the available compatible pipette tips, modified systems demonstrating LESA at smaller sampling areas may be developed in the future.

LESA has been demonstrated for protein analysis from a range of biological substrates. Initial work on LESA MS of dried blood spots revealed its potential for direct detection of variants of haemoglobin [25]. Protein extraction and analysis by LESA MS using denaturing solvent conditions enabled the distinction between multiple Gram-positive and Gram-negative bacteria by direct sampling of the colonies [26]. In that work, top-down MS/MS identification was performed for 40 proteins. Further analysis of dried blood spots has been performed using bottom-up [27] and top-down [28] proteomics workflows. Top-down analysis of blood spots under non-denaturing ‘native’ conditions allowed the detection of heme-bound haemoglobin complexes, most interestingly the heterotetramer, (αβ^2H^)_2_ [29]. Several studies have used LESA for intact protein analysis directly from thin tissue sections. Smaller proteins are often reported; analysis of mouse tissues resulted in the detection of proteins up to ∼20 kDa, including calmodulin (16.8 kDa) and α-globin (15.0 kDa) identified by LESA tandem mass spectrometry (see the section below) [30]. Different proteins may be detected based on tissue storage protocol, as evidenced by the analysis of heat-treated versus fresh frozen tissue [31].

An inherent challenge of *in situ* analysis is the complexity of the resulting sample. To address that challenge, LESA has been coupled to high-field asymmetric waveform ion mobility spectrometry (FAIMS) [32]. FAIMS can improve the signal-to-noise ratio for protein ion signals by filtering out interfering signals arising from other extracted analytes from both tissue [30,31,33] and bacterial colonies [33,34]. Travelling wave ion mobility spectrometry (TWIMS) [35] has been combined with LESA MS [36] to determine the 3D shape of protein ions (in the form of collision cross sections) but its utility as a FAIMS-like filter has yet to be explored. Other ion mobility separation techniques, for example, trapped ion mobility spectrometry (TIMS) [37], may offer similar advantages. A recent study also investigated the ability to derive quantitative measurements of protein concentration directly from tissue during LESA MS experiments [38].

In native MS, information on the solution-phase state of proteins is inferred by the analysis of protein ions in the gas phase [39]. Native MS is achieved by the use of non-denaturing, physiologically mimicking solvent systems, soft ionisation and desolvation conditions and careful optimisation of instrument voltages. Effective desolvation is key for achieving accurate mass measurements and charge state determination [40]. Native MS under optimised conditions preserves protein quaternary structure, the result of delicate non-covalent interactions, allowing the analysis of folded proteins and protein–ligand complexes [40–42]. Native MS is almost universally performed by nanoESI, although other ion sources have been investigated [43–45], and can be combined with LESA by use of an appropriate solvent system. Native LESA MS was demonstrated for the analysis of purified protein complexes exceeding 800 kDa from a glass substrate [46]. Analysis from tissue enabled small, intact proteins to be detected as well as the 64 kDa tetrameric haemoglobin complex [36]. Native LESA MS was recently demonstrated for analysis of proteins and complexes from thin tissue sections on a high mass range Orbitrap mass spectrometer [47]. Proteins exceeding 70 kDa were detected. In that work, a LESA extraction solvent containing the detergent C8E4, often used for native MS of membrane proteins [48], was also considered and proteins up to 47 kDa were detected. The combination of detergent and high mass range MS resulted in richly populated mass spectra, including peaks corresponding to protein complexes such as the 42.6 kDa reactive intermediate deiminase A homotrimer.

## Flow-based techniques

Nano-DESI (Figure 1B) is a continuous flow-based technique that has been reported for high spatial resolution (<20 μm) surface sampling of biological samples [49]. Nano-DESI may be automated, and requires no substantial sample preparation; however, the best performance of the nano-DESI probe has been shown to require technical skill and supplementary apparatus and there is currently no commercial implementation [50].

Despite its name, nano-DESI is more similar to LESA than DESI in terms of analyte extraction mechanism, but differs in that the solvent flows continuously. A liquid microjunction is formed between two fused silica capillaries and the sample surface. Solvent flows from the incident capillary, the material is dissolved from the surface and the sample flows through the second capillary to the mass spectrometer inlet to be ionised by nanoESI. To effectively maintain the liquid microjunction, incremental modifications have been made to nano-DESI ion sources since its inception. These modifications include automatic readjustment of the *z*-axis of the stage for samples with complex topography [49,50,51], and have been demonstrated to provide consistent spatial resolution of up to 10 μm over a period of many hours. This spatial resolution is in the realm of all but the most sophisticated MALDI imaging experiments but without the requirement for matrix deposition [52]. Much of nano-DESI research has focused on lipid analysis, and the highest spatial resolution values are reported for such analytes; however, nano-DESI was combined with light microscopy to detect denatured proteins including haemoglobin, ubiquitin and β-thymosin 4 [53]. The same experimental setup was recently used to profile proteins and peptides in leech tissue [54]. Nano-DESI MSI of proteins up to 15 kDa in healthy and tumour-ridden regions of the mouse brain (spatial resolution ∼200 μm) has also been reported [55]. To date, nano-DESI has not been reported for native protein analysis.

The FlowProbe (Figure 1C, Prosolia, Indianapolis, IN) [56,57] is a commercial system taking advantage of continuous solvent flow and liquid microjunction formation between the probe tip and sample surface. Protein analysis directly from rat brain sections resulted in the detection of 84 multiply charged ion signals, attributable to proteins [58]. FAIMS was used to improve the signal to noise ratio for protein signals. Protein MSI was also performed on mouse brain, allowing detection of small, abundant proteins like ubiquitin (8.6 kDa) directly from tissue [59]. The authors concluded that while raster sampling provided spatial resolution down to 50 μm × 600 μm, this was to the detriment of ion image accuracy. It has also been noted that the polyimide coating of the probe capillaries is subject to swelling as a result of the acetonitrile which constitutes a major component of the most effective denaturing protein sampling solvent blends [59].

Remote, liquid droplet-based sampling has also been demonstrated in the form of the MasSpec Pen. This device is currently designed with sampling location diameters of 1.5–5.0 mm [9]. Negative ion mode analysis of human breast tissue sections enabled the detection of β-thymosin 4 (4.96 kDa) but larger proteins have yet to be reported [9]. Another study [60] focusing on the analysis of intact proteins directly from cell suspensions incorporated online electroporation and electrophoresis into a nanoESI-like setup to release proteins for analysis. This approach enabled the analysis of proteins up to 44 kDa. Electroporation/electrophoresis could be applied to other ion sources discussed above. Another ion source, the open port sampling interface (OPSI) [61] has been demonstrated for recombinant protein standard analysis.

## Desorption techniques

DESI is now routinely applied to the analysis of lipids and metabolites, with recent studies investigating lipidosis within rodent lungs [62], and lipidomic and metabolic profiling of kidney cancers [63,64]. Few studies have focused on the analysis of intact proteins by DESI (Figure 1D) suggesting it remains a considerable challenge. DESI is a relatively mature technique for *in situ* analysis of lipids and the intellectual property rights were recently acquired by Waters Corporation (https://waterscorporation.gcs-web.com/news-releases/news-release-details/waters-bolsters-mass-spectrometry-imaging-portfolio-acquisition), suggesting continued commercial interest in its development. DESI currently requires a level of expertise to operate effectively as there are many parameters to optimise, particularly for *in situ* protein analysis, with only a few examples published to date.

Optimised DESI conditions taking advantage of TWIMS and providing spatial resolution of ∼150 μm for protein analysis were reported recently [65]. TWIMS enabled the separation of the more intense lipid signals from the lower abundance, isobaric protein signals, an approach that will likely be important for many direct tissue analysis implementations. Similarly, the combination of DESI and FAIMS enabled the detection of protein signals from mouse (brain, kidney) and human (ovarian, breast) tissues on Orbitrap mass spectrometers [66]. Ultraviolet photodissociation (UVPD) and collision-induced dissociation (CID) were used for top-down identifications directly by DESI MS. Interestingly, the optimised conditions for both DESI studies report a heated mass spectrometer inlet, but differ in the reported optimal DESI spray voltage (2.5–5.0 kV [65] and 1 kV [66]). Serine dissolved in the DESI solvent reportedly improved protein desalting when analysing standards deposited on a surface [67]. It has also been reported that humidity affects spray stability in negative mode [68]. Clearly, DESI requires further investigation to develop our understanding for protein analysis and this is certainly a barrier to its adoption in routine or high-throughput analysis environments. One study has reported DESI for native protein analysis of purified proteins, successfully ionising large non-covalent complexes such as GroEL_14-mer_ [69]. Insoluble membrane proteins were also analysed by the addition of detergents to the DESI solvent system. Currently, native DESI has not been performed for *in situ* analysis of proteins in tissue.

Tu and Muddiman [70] recently reported that infrared matrix-assisted laser desorption/ionisation (IR-MALDESI) was suitable for intact protein complex analysis. The technique uses an infrared laser to desorb material, rather than a solvent spray, with electrospray post-ionisation. While not demonstrated for *in situ* analysis, IR-MALDESI of holo-myoglobin was demonstrated. IR-MALDESI is already used as an MSI ion source and makes use of water as a matrix, so the application to *in situ* analysis is a logical next step.

Analysis of material by laser ablation/droplet capture was demonstrated in a spatially resolved proteomics strategy [71]. When combined with a nanoPOTS workflow, hundreds of proteins were identified from 50 μm diameter laser-dissected tissue samples; however, the workflow inherently relies on enzymatic digestion and liquid chromatography separation to achieve these numbers.

## Intact protein identification

**Figure 2.**
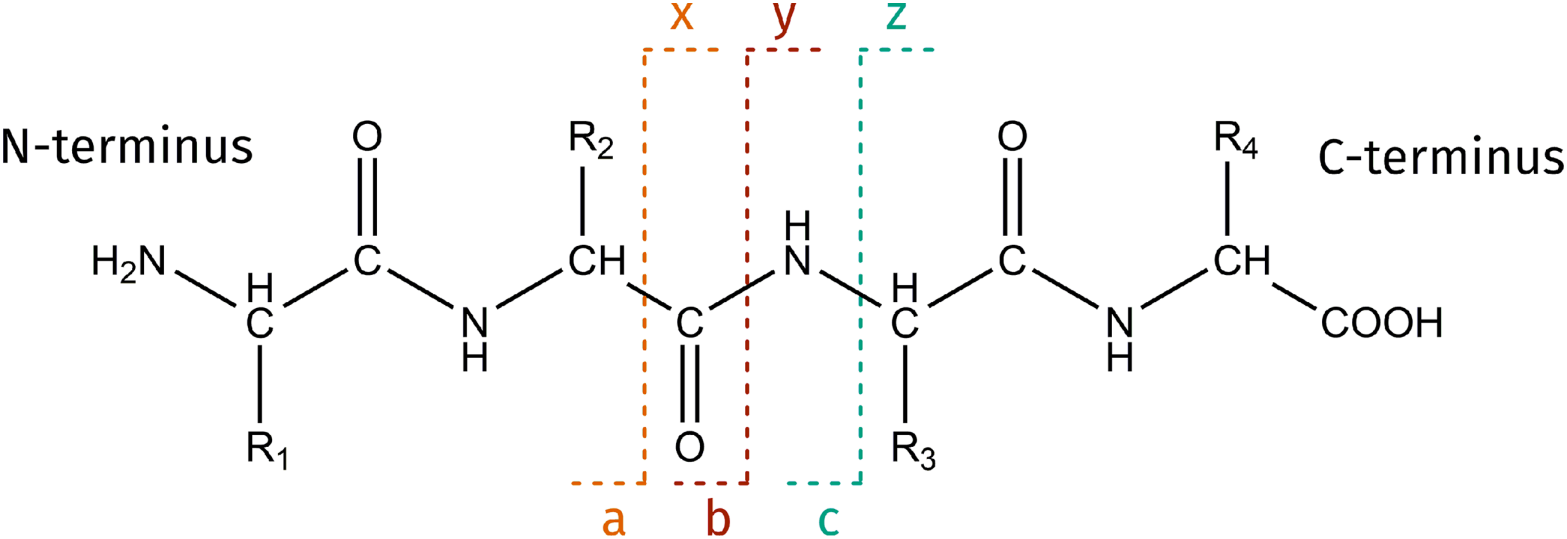
Annotation of peptide fragment ions according to [73]. The a/b/c ions are N-terminal fragments, while x/y/z fragments are C-terminal. CID and HCD predominantly result in b and y ions; electron-mediated techniques, c and z ions; and UVPD, a complex mixture of a/b/c, x/y/z.

Intact protein analysis is dependent on a top-down approach to protein identification. Tandem mass spectrometry (MS/MS) techniques may be used individually, or in combination, to fragment intact protein ions within the mass spectrometer. The fragment ions provide sequence information for the protein [72]. The most commonly available MS/MS method is CID. Protein ions are collided with a neutral gas, typically nitrogen or argon. The energy from these collisions is redistributed throughout the ion and ultimately results in peptide bond cleavage throughout the protein backbone. While it is generally less efficient for larger proteins, CID produces extremely predictable fragmentation patterns predominantly featuring b and y ions (see Figure 2) [72,73]. A variant of CID found on Orbitrap mass spectrometers is higher-energy collision dissociation (HCD) [74]. HCD produces similar fragment ion spectra to CID. *In situ* top-down identification of proteins has been reported for FlowProbe and CID [58], nano-DESI with CID [54], DESI with CID [66] and LESA with CID [26,75] and HCD [34].

Electron-mediated techniques such as electron transfer dissociation (ETD) [76] and electron capture dissociation (ECD) [77] produce fragment ion spectra featuring c and z ions (see Figure 2), resulting from fragmentation of N-Cα bonds in the polypeptide chain. Since ETD and ECD are reduction processes [78], multiply charged precursor ions are a necessity and this is an advantage if using an ion source that produces multiply charged ions. For large proteins, ETD and ECD tend to produce greater sequence coverage than solely collision-based techniques since fragmentation occurs randomly throughout the polypeptide chain; this is particularly apparent with supplemental activation [79]. Importantly, these techniques are able to retain labile PTMs such as phosphorylation and glycosylation [80–83]. ETD and ECD are also able to cleave bonds in the protein backbone without dissociation of non-covalent interactions, e.g. salt bridges, in native MS [84,85]. The electron-mediated technique, electron ionisation dissociation (EID) also produces a wealth of a/b/c and x/y/z fragment ions from intact proteins [86]. LESA coupled to liquid chromatography separation and top-down ETD MS/MS has been used to provide protein identification information to complement separately collected high-resolution MALDI imaging data from mouse pup sections.[23] LESA ETD MS/MS also enabled the identification of two variants of fatty acid-binding protein in human liver [75].

UVPD involves the absorbance of UV photons by protein ions, causing a transition to an excited electronic state and subsequent fragmentation. UVPD spectra are richly populated with a/b/c and x/y/z ions due to fragmentation occurring throughout the polypeptide chain. UVPD is still a relatively niche MS/MS method, with limited commercial availability, but research continues to demonstrate its utility for intact protein analysis [66,87,88]. Currently, only DESI has been demonstrated for *in situ* protein sequencing by UVPD [66].

Manual interpretation of top-down MS/MS data is an intensive process and is thus ideally performed by bioinformatics tools capable of automatic, unsupervised identification. The intact mass of the protein is usually determined by deconvolution of the charge state distribution of the multiply charged precursor ions, or by direct calculation if an accurate mass and charge state can be determined. Assuming MS/MS spectra of sufficient quality are obtainable by fragmenting the precursor ions, automatic peak detection, accurate mass measurement and charge state assignment are powerful for unambiguous identification. Many of the same considerations as bottom-up proteomics database searching also apply to top-down searches; e.g. keeping the database focused to the appropriate proteomes to minimise search time, applying scoring to assess identification probability. Top-down MS/MS must also consider labile PTMs and bound ligands (under native conditions). Tools designed for top-down MS/MS interpretation and database searching include ProSightPC [89] (now marketed by Thermo Scientific), TopPIC [90], SpectroGene [91], MSPathFinder [92], pTop [93] and TopMG [94]. ProsightPC has been integrated with Proteome Discoverer (Thermo Scientific) and considering the most recent Orbitrap MS instrumental developments bring improvements to intact protein analysis, indicates substantial commercial interest in developing top-down MS/MS into a routine procedure.

## Perspectives

- *In situ* analysis of intact proteins offers the opportunity for direct analysis of proteins with reduced sample preparation compared with LC–MS/MS methods. Since spatial information may also be retained, it is possible to assign location, conformation and mass from a single experiment, i.e. gain a wealth of information for the investigation of protein function.
- The challenge of protein dynamic range within cells remains considerable for direct analysis techniques and suggests why many of the studies accounted for here report the same proteins. *In situ* protein analysis must progress beyond these to become a more useful tool. Ion mobility separation has already demonstrated its value for improving the numbers of detected proteins and will undoubtedly continue to be useful as the field progresses.
- Much of the potential value of intact protein mass spectrometry lies in the analysis of large protein complexes and low abundance species to aid our understanding of diseases and their treatments. Further development of the current tools and methods, and perhaps even entirely novel techniques, will be needed to achieve *in situ* analysis of these species and realise that goal.

## Abbreviations

CID: collision-induced dissociation
DESI: desorption electrospray ionisation
ECD: electron capture dissociation
EID: electron ionisation dissociation
ESI: electrospray ionisation
ETD: electron transfer dissociation
FAIMS: high-field asymmetric waveform ion mobility spectrometry
HCD: higher-energy collisional dissociation
IMS: ion mobility spectrometry
IRMPD: infrared multiphoton dissociation
LESA: liquid extraction surface analysis
LMJ-SS: liquid microjunction-surface analysis
*m*/*z*: mass-to-charge ratio
MALDI: matrix-assisted laser desorption/ionisation
MS: mass spectrometry
MS/MS: tandem mass spectrometry
MSI: mass spectrometry imaging
nano-DESI: nanospray desorption electrospray ionisation
nanoESI: nanoelectrospray ionisation
OPSI: open port sampling interface
TIMS: trapped ion mobility spectrometry
TWIMS: travelling wave ion mobilty spectrometry
UVPD: ultraviolet photodissociation

## Open Access

Open access for this article was enabled by the participation of University of Birmingham in an all-inclusive *Read & Publish* pilot with Portland Press and the Biochemical Society under a transformative agreement with JISC.
